# Successful hemostasis and subsequent cannulation using a self-assembling peptide hydrogel for bleeding after precut sphincterotomy

**DOI:** 10.1055/a-2085-0838

**Published:** 2023-05-26

**Authors:** Tadahisa Inoue, Mayu Ibusuki, Rena Kitano, Yuji Kobayashi, Kiyoaki Ito, Masashi Yoneda

**Affiliations:** Department of Gastroenterology, Aichi Medical University, Aichi, Japan

Precut techniques, such as needle-knife sphincterotomy and transpancreatic sphincterotomy, are useful as salvage techniques for cases in which biliary cannulation is difficult 1 . If massive bleeding occurs during precutting, various methods may be used to achieve hemostasis, including epinephrine injection, thermal cautery, and clipping. However, all of the above may affect the duodenal papilla, complicating subsequent cannulation. Additionally, the coagulum and hematoma resulting from such an event obscure the papilla, which also impedes cannulation. A novel, self-assembling peptide hydrogel (PuraStat; 3-D Matrix, Tokyo, Japan) was recently developed as a hemostatic agent 2 3 4 . We present a case in which this hydrogel was useful for hemostasis and subsequent cannulation following massive bleeding during precut sphincterotomy.

A 73-year-old man developed obstructive jaundice with cholangitis owing to bile duct stricture. Endoscopic retrograde cholangiography was performed, but biliary cannulation was unsuccessful. As the pancreatic duct could be cannulated, we attempted the double-guidewire technique. However, as the difficulties persisted, we decided to conduct precut transpancreatic sphincterotomy.

During the precutting, bleeding occurred, which rapidly worsened, and a clear endoscopic view could not be obtained owing to the coagulum. Therefore, a gel-filled catheter was inserted into the coagulum and its tip positioned at the assumed origin of the bleed. A large volume (3 mL) of the peptide hydrogel was injected to achieve hemostasis and to shift the coagulum. Consequently, complete hemostasis was achieved, and the incision surface became visible through the hydrogel ( Fig. 1 , Video 1 ). Finally, biliary cannulation was successfully performed with the double-guidewire technique across the hydrogel, followed by plastic stent placement. No adverse events, including rebleeding and pancreatitis, were observed after the procedure.

**Fig. 1:**
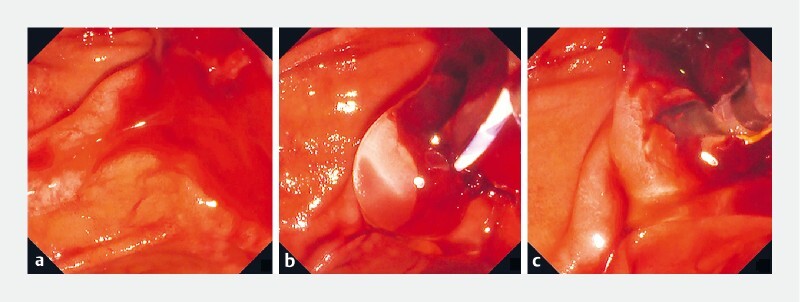
Endoscopic views. **a** Bleeding occurred during precut transpancreatic sphincterotomy and rapidly worsened, impeding the endoscopic view of the duodenal papilla owing to coagulum. **b** A large volume of the self-assembling peptide hydrogel was injected to achieve hemostasis and to shift the coagulum. **c** Complete hemostasis was achieved, and the incision surface became visible through the hydrogel. We achieved biliary cannulation with the double-guidewire technique across the hydrogel, with no adverse events.

**Video 1** Successful hemostasis and subsequent cannulation using a novel, self-assembling peptide hydrogel for bleeding after precut sphincterotomy.


The PuraStat hydrogel easily stopped bleeding in this case without affecting the papilla, and simultaneously pushed the accumulated coagulum aside. Therefore, it may be a useful treatment option for hemostasis after precut sphincterotomy-induced bleeding.

Endoscopy_UCTN_Code_CPL_1AK_2AC

## Notes

Endoscopy E-Videos

https://eref.thieme.de/e-videos
*E-Videos* is an open access online section of the journal *Endoscopy* , reporting on interesting cases and new techniques in gastroenterological endoscopy. All papers include a high-quality video and are published with a Creative Commons CC-BY license. Endoscopy E-Videos qualify for HINARI discounts and waivers and eligibility is automatically checked during the submission process. We grant 100% waivers to articles whose corresponding authors are based in Group A countries and 50% waivers to those who are based in Group B countries as classified by Research4Life (see: https://www.research4life.org/access/eligibility/ ). This section has its own submission website at https://mc.manuscriptcentral.com/e-videos
